# Environmental Sanitation Crisis: More than just a health issue

**DOI:** 10.4137/EHI.S1047

**Published:** 2008-09-16

**Authors:** Peter A. Harvey

**Affiliations:** Chief, Water and Environmental Sanitation, UNICEF, Zambia, Associate, Water, Engineering Development Centre, Loughborough University, U.K

**Keywords:** developing countries, environmental sanitation, preventive health

## Abstract

The global environmental sanitation crisis cannot be denied: well over a century after the sanitary revolution in 19th century Europe, 40% of the world’s population still lacks access to improved sanitation. Important lessons from the past must be applied today if the crisis is to be averted. Sanitation has suffered from a lack of prioritization for as long as it has remained the poor relation to water supply. The International Year of Sanitation 2008 provides an opportunity to separate the two and give sanitation the emphasis it requires. The economic argument for sanitation must be articulated and non-health incentives for improved sanitation exploited. Environmental sanitation results in a multitude of socio-economic benefits and can contribute positively to all the Millennium Development Goals. Community-led bottom-up approaches, rather than supply-led or technology-driven approaches, are most effective in increasing and sustaining access to sanitation but need to be implemented at scale. Targeted strategies for urban and school sanitation are also required. Evidence-based advocacy can help develop the political will that is now needed to ensure sufficient public sector investment, leadership, legislation and regulation to ensure that the fundamental human right of access to sanitation is realized.

## Introduction

In a recent poll of readers of the British Medical Journal ‘the sanitary revolution’ of introducing piped water and waterborne sewerage to people’s homes in 19th Century Europe was voted the most important medical milestone since 1840, beating even the discovery of antibiotics and the development of anesthesia (BMJ, 2007). Well over a century after this breakthrough in industrialized nations, it is estimated that 2.5 billion people, 40% of the world’s population, still lack access to basic sanitation (UNICEF/WHO, 2008). One of the Millennium Development Goal (MDG) targets is to halve the proportion of people without access to sanitation by the year 2015. Estimates by the Joint Monitoring Program of the United Nations Children’s Fund (UNICEF) and the World Health Organization (WHO) indicate that at the current rate of progress the world will miss the target by over 700 million people. This will mean that approximately 2 billion people will still lack access in 2015. In sub-Saharan Africa this modest target, which still denies 50% of people in need of this basic human right, will not be met until 2072 if the current rate continues (Lancet, 2008). Meanwhile, diarrhea remains the second highest single cause of child mortality worldwide (Bryce et al. 2005). In 2006, the United Nations General Assembly designated 2008 as the International Year of Sanitation (IYS), recognizing that access to sanitation is vital to ensuring health, dignity and sustainable social and economic development for the world’s poorest citizens.

## Why is Sanitation Coverage so Low?

There is no doubt that sanitation has suffered from a lack of prioritization in development plans, particularly when compared to water supply. The coupling of water and sanitation is based primarily on the need for water to supply waterborne sewerage systems in industrialized nations. In many developing nations dry onsite sanitation systems are the norm and hence there is no direct requirement for water (other than for personal and domestic hygiene). However, the prevalent link between the two components often means that sanitation is seen as a less important ‘add-on’ to water supply programs. The phrase ‘water is life’ is commonly cited and the importance of water is recognized by all. The need for sanitation and the subsequent multiple benefits are normally less well understood, and hence sanitation is given less attention than it deserves by governments and development partners. The story of Dr. John Snow’s discovery of the link between water from a hand pump in Broad Street and the cholera epidemic in London in 1854 has been recounted numerous times and consequently the link between water quality and diarrheal disease is well established. What is less well known, however, is the discovery by Reverend Whitehead that the well became contaminated from the excreta of a cholera patient which was disposed of in a cesspool very close to the well and that the brickwork of the drain and cesspool were highly defective (Black and Fawcett, 2008). Thus, despite the emphasis on water, poor sanitation was in fact the primary cause of the epidemic. The inequitable partnership between water and sanitation suggests that the ‘sacred’ link between the two at the heart of many development policies should be broken. Sanitation should be addressed separately from water supply, at least in terms of policy, strategy and funding, to ensure that it receives sufficient emphasis and prioritization.

## The Economic Argument for Sanitation

The improvements in sanitation in 19th Century Europe were motivated primarily by economic factors and the public good rather than altruistic objectives to improve the health of poor urban dwellers. The required political will developed only when it was recognized that high morbidity rates among skilled laborers were hindering industrial and economic progress, and that diseases among the urban poor might threaten the better-off. Just as the drivers behind the European ‘sanitary revolution’ were economic rather than altruistic, the economic argument needs to be made for the global sanitation revolution that is required today. A recent study by the World Health Organization estimates an economic return of $9.1 on every $1 invested in sanitation (Hutton et al. 2007).

Environmental sanitation encompasses not just excreta disposal and management, but also solid waste management, drainage and hygiene practices. Improved environmental sanitation affects positively a wide range of development indicators. In fact, it is clear that sanitation has an impact on each of the MDGs:

- *MDG 1: Eradicate Extreme Poverty and Hunger*—Improved sanitation leads to a reduction in diarrheal morbidity of 37.5% (Esrey, 1996), and hence more productive days are gained for income generation activities, agriculture and education.
- *MDG 2: Achieve Universal Primary Education*—The provision of sanitation facilities in primary schools leads to increased school enrollment and attendance and reduced drop-out rates (especially among girls). Reduced diarrheal disease also leads to an increase in school attendance days.
- *MDG 3: Promote Gender Equality and Empower Women*—The provision of gender appropriate sanitation and hygiene facilities in schools supports the educational advancement of girls, and community sanitation programs encourage the participation of women.
- *MDG 4: Reduce Child Mortality*—Since diarrhea is the second highest single cause of child mortality, access to improved sanitation and hygiene leads to significant reductions in child mortality. It is estimated that there are 1.5 million diarrheal-related deaths per year of children under five years old (UNICEF 2006).
- *MDG 5: Improve Maternal Health*—Ensuring access to adequate sanitation and supporting improved hygiene practices helps to reduce diarrheal disease and other related maternal health issues.
- *MDG 6: Combat HIV/AIDS, Malaria, and Other Diseases*—Improved drainage and solid waste management reduce breeding grounds for mosquitoes, and improved sanitation and hygiene for people with HIV and AIDS help prevent health complications.
- *MDG 7: Ensure Environmental Sustainability*—Improved excreta disposal, solid waste management and drainage protects the quality of water resources and creates a clean and safe environment; effective sanitation provision will halve the proportion of people without access to sanitation by the year 2015.
- *MDG 8: Develop a Global Partnership for Development*—The International Year of Sanitation is a good starting point to develop global partnerships for prioritizing sanitation and linking it with other sector goals.

The cost of meeting the water and sanitation MDG targets per year until 2015 is estimated to be between U.S. $9.5 billion (Hutton and Haller, 2004) and U.S. $11.3 billion (UNICEF/WHO 2006). Given the magnitude of the required investment it is essential that the multitude of economic and social benefits of improved environmental sanitation are well understood by decision-makers.

## New Approaches to Sanitation: What Works and What Doesn’t?

The supply-led approaches of the past, whereby toilets were constructed by external support agencies, whether or not the recipient community recognized the need for them, have proved to be unsuccessful. Such toilets were often not used or they were not sustained once they had reached the end of their lifespan. With increased awareness of the scale of the global sanitation challenge, innovative strategies are now being developed to accelerate sanitation coverage. One of these approaches is Community-Led Total Sanitation (CLTS), first developed in Bangladesh in 1999 and now being implemented in an increasing number of countries in Asia, Africa and Latin America. CLTS is a powerful social mobilization tool to achieve universal access to sanitation. It facilitates a process of empowering local communities to stop open defecation and to build and use toilets themselves without the support of any hardware subsidy. It is based on the concept of self-respect rather than on standards or health. In rural communities in which open defecation has been practiced for generations people are not easily persuaded to change their practices on the basis of health education alone. CLTS engenders a sense of disgust regarding unsanitary behaviors and uses non-health incentives for sanitation uptake, such as status, esteem, peer pressure and economics. It also focuses on the public good and overall well being of the community. The costs of lack of sanitation, resulting from healthcare needs and loss of productive time, are compared to that of constructing a simple family toilet and the comparative economic advantages soon become apparent to people. The importance of dignity and privacy are also emphasized, and CLTS is commonly used as an entry point for other community development and economic empowerment initiatives.

CLTS was recently piloted for the fist time in Zambia and has seen significant and rapid success. Here, Traditional Leaders, Environmental Health Technicians, Ward Councilors and Civic Leaders were trained as CLTS facilitators and then visited their respective rural communities where they engaged community members through the CLTS triggering process. Subsequently each community resolved to put an end to open defecation and formed a sanitation action group to lead the process of toilet construction and behavior change in each village. The program saw an increase in sanitation coverage from 23% to 88% in just two months (Harvey and Mukosha, 2008). This coverage was based on the ratio of the number of toilets to the number of households. In the majority of communities coverage increased from less than 20% to more than 90% in this time period. Such rapid increases in sanitation provision have been unheard of in the past and suggest that the approach has the potential to significantly decrease the proportion of people without access to improved sanitation.

The success of the CLTS approach hinges on the fact that communities are able to construct toilets for themselves without dependency on external agencies or ‘handouts’. Such transitions from dependency to self-sufficiency have also occurred in successful agricultural development programs in southern Asia (D’Monte, 2006). A similar approach to CLTS is Total Sanitation in India, however, this includes the provision of financial incentives to ‘open defecation free’ villages which may not be sustainable. The beauty of the CLTS concept is that communities are enabled to see and feel the importance of sanitation and consequently they become motivated to do something about it themselves. They are not driven by incentives or rewards but by the desire to live in communities in which everyone uses a toilet and does not defecate in the open. It is important that such successes are critically evaluated, documented and disseminated, so that lessons learned from the process can be applied elsewhere. The financial requirements of such programs must also be evaluated to determine budgetary allocations.

Over the past few decades innovative sanitation technologies have also been promoted in low-income countries. One of these, ecological sanitation, is often promoted by Western development agencies. This entails the use of ecological toilets in which human waste is dehydrated or composted for sufficient time so that it can be reused as a soil conditioner for agricultural use. Given increasing concerns over the potential global shortage of phosphorous and the rising price of fertilizers, it makes economic and environmental sense to reuse human excreta in this way. However, such projects are often supply-led, rather than responding to felt needs of communities, and they are not likely to be successful unless communities fully understand the benefits and are able to exploit these. One important obstacle that must be overcome is the necessary handling of semi-decomposed human waste and the social, cultural and health factors that may hinder this. The public health concerns and lack of profitability that led to the abandonment of the reuse of human excreta in much of Europe should perhaps be considered by today’s enthusiasts for ecological sanitation (Black and Fawcett, 2008). Other technological sanitation solutions such as toilets that use human waste for biogas production have been widely adopted in some parts of Asia but also require extensive community sensitization and may not be appropriate in many low-income countries.

Given the increasing urbanization of the developing world it is important that specific strategies are developed for urban and peri-urban sanitation. While in most countries sanitation coverage is significantly higher in urban areas than rural areas, growing inequality within cities divides formal areas and informal settlements (WSP, 2008). Given the high population densities in the latter, strategies may need to consider shared sanitation facilities even though these are not currently included in the MDG definition of ‘improved sanitation’ (UNICEF/WHO, 2008).

School sanitation and hygiene programs, as championed by UNICEF and Non-Governmental Organizations (NGOs), also play key roles in improving environmental sanitation and enhancing education performance. By including these aspects in school curricular generational changes in attitudes and practices may be achieved. In Burkina Faso, Colombia, Nepal, Nicaragua, Vietnam and Zambia successful approaches and strategies have been developed for the overall planning of School Sanitation and Hygiene Education (SSHE), life-skills based hygiene education, child-friendly designs of water, sanitation and hygiene facilities, participation of children and young people, and monitoring and evaluation (UNICEF/IRC, 2006). Such programs have also been successful in reaching out from schools to surrounding communities to promote improved environmental sanitation and hygiene behavior.

One final lesson from the European ‘sanitary revolution’ is the need for public sector leadership, legislation and regulation. While the private sector can provide different technical options, sanitation coverage will not increase unless there is strong government commitment and increased public sector investment, and unless appropriate sanitation strategies are developed and implemented.

## What Next?

In order to avert the current global sanitation crisis it is essential that sanitation be finally given the attention that it deserves. The first step in this, and the opportunity provided by the International Year of Sanitation, is to unlink water and sanitation. This healthy divorce will mean that sanitation need no longer be the poor relation and that all relevant stakeholders might finally take it seriously. Given the increased global emphasis on the environment, the environmental benefits of sanitation, such as good quality water resources, reduced environmental degradation and increased energy efficiency through waste recycling, should also be maximized.

Community-led bottom-up approaches should be promoted and implemented at scale rather than sticking to small-scale projects. Supply-led or technology-driven approaches, particularly those originating from outsiders, must be viewed with caution and the sustainability and applicability of such initiatives should always be questioned. Sanitation is predominantly a social issue not a technical one.

Implementation approaches need to be critically analyzed and research gaps filled in order to provide the necessary evidence to advocate for preventive environmental health measures rather than relying on curative interventions. The multiple links between sanitation and non-health benefits should also be investigated in detail. The sanitation sector, in general, has been relatively week in generating the evidence needed to convince policy-makers to prioritize sanitation. Perhaps this is because to date there has not been such a sector. If there ever was a time to establish the Environmental Sanitation Sector, that time is now.
